# Surveillance of Airborne Adenovirus and *Mycoplasma pneumoniae* in a Hospital Pediatric Department

**DOI:** 10.1371/journal.pone.0033974

**Published:** 2012-03-21

**Authors:** Gwo-Hwa Wan, Chung-Guei Huang, Yhu-Chering Huang, Ju-Ping Huang, Su-Li Yang, Tzou-Yien Lin, Kuo-Chien Tsao

**Affiliations:** 1 Department of Respiratory Therapy, College of Medicine, Chang Gung University, Tao-Yuan, Taiwan; 2 Department of Laboratory Medicine, Chang Gung Memorial Hospital, Tao-Yuan, Taiwan; 3 Department of Biotechnology and Laboratory Science, Research Center for Emerging Viral Infections, Chang Gung University, Tao-Yuan, Taiwan; 4 Division of Pediatric Infectious Diseases, Chang Gung Memorial Hospital, Tao-Yuan, Taiwan; 5 College of Medicine, Chang Gung University, Tao-Yuan, Taiwan; University of Hong Kong, Hong Kong

## Abstract

This investigation evaluated the distributions of airborne adenovirus and *Mycoplasma pneumoniae* in public areas in the pediatric department of Children's Hospital in northern Taiwan. The airborne viral and bacterial concentrations were evaluated twice a week for a year using filter sampling with an airflow rate of 12 liters per minute for eight hours in the pediatric outpatient department and 24 hours in the pediatric emergency room. Real-time polymerase chain reaction assays were conducted for analysis. Approximately 18% of the air samples from the pediatric emergency room were found to contain adenovirus. Approximately forty-six percent of the air samples from the pediatric outpatient department contained *Mycoplasma pneumoniae* DNA products. High detection rates of airborne adenovirus DNA were obtained in July and August in the pediatric public areas. Airborne *Mycoplasma pneumoniae* was detected only in July in the pediatric emergency room and the peak levels were found from August to January in the pediatric outpatient department. Airborne particles that contained adenovirus and *Mycoplasma pneumoniae* were the most prevalent in the pediatric public areas. The potential relationship between these airborne viral/bacterial particles and human infection should be examined further.

Keywords: adenovirus; *Mycoplasma pneumoniae*; filter sampling; real-time polymerase chain reaction; hospital; pediatric public areas.

## Introduction

Viral respiratory infection is the most common infectious disease. The viruses, such as enterovirus, influenza virus, and adenovirus, were most often isolated from the pediatric outpatients [1]. Adenovirus causes acute respiratory tract infections in children younger than 5 years old. *Mycoplasma pneumoniae* is a common causative pathogen of community-acquired pneumonia (CAP) in children and young adults. In Taiwan, *Mycoplasma pneumoniae* was reported to account for 20–30% of CAP cases [2], [3]. Airborne viruses are known to be associated with larger particles and aggregate in nature [4]. Most aerosolized droplet nuclei are between 0.58 and 5.42 µm in diameter [5]. One investigation found 53% of detectable influenza virus particles in the respiratory aerosol fraction (1–4 µm) [6].

Three primary factors - the virus concentration in the air, the collection efficiency of the air sampler, and the sensitivity of the analytical assay to the target pathogen - determine the probability of detecting airborne viral pathogens [7]. Previous studies have found that sampling devices such as the all-glass impinger 30, the SKC BioSampler and a frit bubbler are unsuited to collect viral particles [8]. Filtration was used to collect airborne particles that contain viruses, including rhinovirus [9], [10], severe acute respiratory syndrome (SARS) coronavirus [11]–[13], RSV [14], enterovirus, adenovirus, and influenza A virus [15]. PCR analysis amplifies nucleic acids exponentially, and is particularly sensitive to airborne infectious agents, such as rhinovirus [9], [10], RSV [14], enterovirus, adenovirus, influenza A virus [15], SARS coronavirus [11]–[13], and measles virus [16]. No standard protocol had been established for collecting and detecting specific viral particles in the air.

Until recently, few studies have examined the long-term distributions of airborne viral and bacterial aerosols in health-care settings in the subtropical region of Taiwan using filter sampling with pathogen-specific PCR assays. Accordingly, in this investigation, airborne adenovirus and bacterial (*Mycoplasma pneumoniae*) concentrations at the pediatric emergency room and pediatric outpatient department of the Children's Hospital in northern Taiwan were evaluated for one year.

## Materials and Methods

### Sampling location

A medical center, Chang Gung Memorial Hospital with 3,500 beds located in north Taiwan, was selected in this study. This hospital contains many departments (such as department of medicine, department of surgery, department of pediatrics, department of gynecology and obstetric, department of Chinese medicine, etc.). The sampling site was the pediatric public areas including the pediatric emergency room and the pediatric outpatient department. In the sampling periods, the air in the pediatric public areas was conditioned without heating. No local source of industrial air pollution was close to the hospital. The study protocol was approved by the institutional review board of the hospital.

### Air Sampling

Air samplers were placed in the center of the pediatric outpatient department and the pediatric emergency room for 8 hours (from 9 am to 5 pm) and 24 hours, respectively, randomly selected twice weekly (Monday to Saturday) for a year (February 2009 to January 2010). Sampling was performed 1.2–1.5 m above the floor, in the human breathing zone. The indoor air was filtered through a closed-face, three-piece disposable plastic cassette with a 0.2 µm polytetrafluoroethylene filter at an airflow rate of 12 L/min (LPM) for bioaerosol sampling. A total of 186 filter samples (93 samples from the pediatric outpatient department and 93 samples from the pediatric emergency room) were collected and the filters were immediately stored at −70°C until they were analyzed.

The air quality indices (air temperature, relative humidity (RH), carbon dioxide (CO_2_) and suspended particular matter) in the pediatric public areas of the hospital were evaluated during the study period. The indoor temperature, RH and CO_2_ concentration were determined using a digital psychrometer (TSI incorporated 500 Cardigan Road, Shoreview, MN 55126, USA). The portable DUSTcheck monitor (Grimm, model 1.108, Germany) was used to measure the mass concentrations of particulate matters.

### Preparation of filter sample extracts and DNA extraction

One thousand and five hundred microliters aliquots of phosphate-buffered saline (PBS) were pipetted into 60 mm diameter petri dishes that each contained a filter, and these petri dishes were then placed on an orbital tabletop shaker at a speed of 150 rpm for 60 minutes at room temperature. All samples were stored at −80°C until DNAs were extracted.

The DNA was extracted using the Qiagen DNA mini kit (Qiagen, Hilden, Germany) according to the manufacturer's directions. Briefly, 500 µL of the sample was extracted and the nucleic acid was eluted in a final volume of 30 µL. All eluted samples were stored at −80°C until analysis. In the negative controls, sterile distilled water was added in place of the samples.

### Real-time quantitative PCR assay

Table 1 presented the sequences of the primers and probes for adenovirus and *Mycoplasma pneumoniae*. The adenovirus and *Mycoplasma pneumoniae* in the filter samples were quantitatively measured as previously described [17]. Concentrations of calibration standards ranged from 10 to 10^6^ copies/µL for adenovirus and 10 to 10^4^ copies/µL for *Mycoplasma pneumoniae*. For the tested adenovirus and *Mycoplasma pneumoniae*, the known amounts of plasmid cDNA yielded cycle threshold (Ct) values ranging between 19.46 and 36.58 cycles. The correlation coefficient (r) values of the calibration curves were 0.99 for adenovirus and *Mycoplasma pneumoniae*.

**Table 1:** The sequences of primers and probes of adenovirus and *Mycoplasma Pneumoniae.*

Oligo Names	Sequences(5′→3′)	Genes	Reference
**Adenovirus**		Hex	-
Ad-s	GCCCCAGTGGGCTTACATGCACATC		
Ad-as	ATTGAAGTAGGTGTCTGTGGCGCGGG		
Ad-probe	FAM-AACTGCACCAGACCCGGGCTCAGGTACTCC-TAMRA		
***Mycoplasma pneumoniae***		P1	17
MP-TM1	CCAACCAAACAACAACGTTCA		
MP-TM2	ACCTTGACTGGAGGCCGTTA		
MP-probe	VIC-ATCCGAATAACGGTGACTT-MGB		

### Statistical analysis

SPSS (Statistical Package for Social Science) version 13.0 (SPSS Inc., Chicago, USA) was used for the statistical analyses. The level of significance was set to 0.05. The figures were constructed using GraphPad Prism 5.0 software. A two-sample *t* test was used to assess the differences of detection rates between adenovirus and *Mycoplasma pneumoniae* in the pediatric emergency room and pediatric outpatient department, respectively.

## Results

During the study period, the air temperatures and relative humidity were 16.1–26.6°C and 42.5–77.7%, respectively, in the pediatric emergency room, and 13.7–24.8°C and 50.4–84.7% in the pediatric outpatient department. The CO_2_ concentrations were 404–704 ppm in the pediatric emergency room and 372–2,575 ppm in the pediatric outpatient department. The mean concentrations of ≦10 µm (PM_10_) and ≦1 µm (PM_1_) particles were 8.9 µg/m^3^ and 17.0 µg/m^3^, respectively, in the pediatric emergency room, and approximately 105 µg/m^3^ and 167 µg/m^3^, respectively, in the pediatric outpatient department.

**Figure 1:**
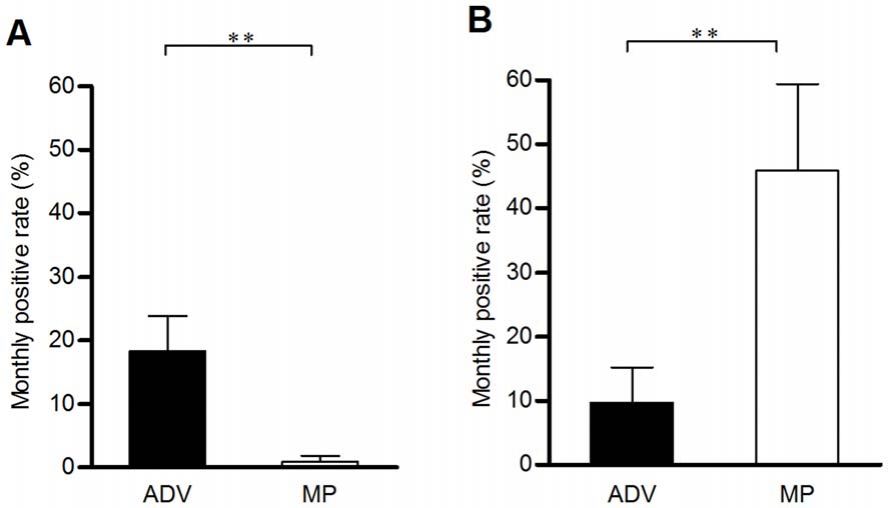
Airborne adenovirus and *Mycoplasma pneumoniae* in the pediatric public areas. The monthly positive rates (mean±se) of airborne particles containing adenovirus (ADV) and *Mycoplasma pneumoniae* (MP) in the pediatric emergency room (ER) (A) and pediatric outpatient department (OPD) (B). **: *p*<0.01.

Filter samples without airflow passage were selected as negative controls. The adenovirus and *Mycoplasma pneumoniae* were not found in the blank filters. Also, PCR positive rates of the positive controls containing adenovirus or *Mycoplasma pneumoniae* in the sampling filters are 100%. The monthly detection rate (18.3%) of the adenovirus in the air samples of the pediatric emergency room of the medical center was clearly higher than that of *Mycoplasma pneumoniae* (0.9% of the air samples) (*p* = 0.009) (Fig. 1). Around 45.8% of the air samples from the pediatric outpatient department contained *Mycoplasma pneumoniae*. Other pathogen, adenovirus (9.8% of the air samples), was also found in the pediatric outpatient department.

**Figure 2:**
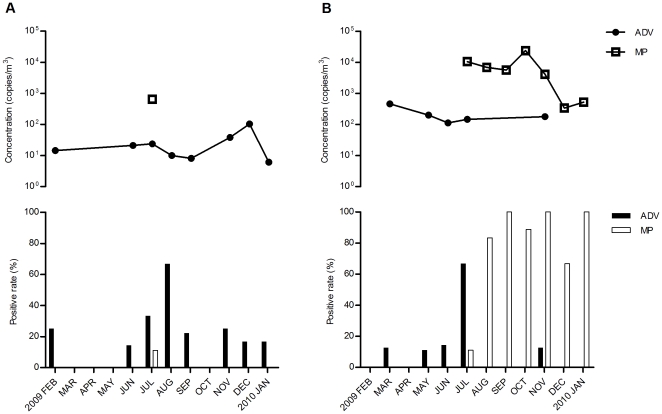
Monthly distributions of airborne ADV and MP in the pediatric ER (A) and OPD (B). Monthly distributions of the detection rates of airborne ADV and MP in the filter samples and the median concentration distributions of ADV and MP in the air of the pediatric public areas.

In this study, airborne particles that contained adenovirus were detected in the pediatric emergency room throughout the year except in March to May, and October (Fig. 2A). Detection rates of airborne adenovirus DNA products in the filter samples in the pediatric emergency room were high in July (33.3%) and August (66.7%). The airborne *Mycoplasma pneumoniae* DNA products were detected in the pediatric emergency room only in July (11.1% of the air samples). The airborne particles that contained adenovirus concentrations ranged between <10 copies/m^3^ and 104 copies/m^3^ in the pediatric emergency room. The median concentration of airborne adenovirus DNA was highest in December. Only one air sample in the pediatric emergency room was positive for *Mycoplasma pneumoniae* DNA product (645 copies/m^3^).

The detection rate of adenovirus-containing particles in the air samples in the pediatric outpatient department peaked in July (66.7%) (Fig. 2B). However, airborne particles that contained *Mycoplasma pneumoniae* were found in the pediatric outpatient department throughout the year except in February to June. High detection rates of airborne *Mycoplasma pneumoniae* DNA products in the filter samples were found in August (83.3%), September (100%), October (88.9%), and November (100%), and January (100%). The detected concentrations of adenovirus in the air in the pediatric outpatient department ranged from 48.4 copies/m^3^ to 461 copies/m^3^, and the median concentration was highest in March. The concentrations of *Mycoplasma pneumoniae* in airborne particles ranged between 114 copies/m^3^ and 9.9×10^4^ copies/m^3^ in the pediatric outpatient department. The median concentration of *Mycoplasma pneumoniae* DNA product in the air of the pediatric outpatient department was highest in October.

## Discussion

This study is the first to examine the long-term variation of the distribution of airborne adenoviral particles and *Mycoplasma pneumoniae* in the pediatric emergency room and pediatric outpatient department of the Children's Hospital in Taiwan. The detection rate of airborne adenovirus DNA products in the pediatric emergency room clearly exceeded those of *Mycoplasma pneumoniae* DNA products. Airborne *Mycoplasma pneumoniae* DNA products were detected in around 46% of samples from the pediatric outpatient department of the hospital. The distribution of microbial species in the pediatric public areas warrants further attention from the department of environmental safety and health to reduce the risk of microbial exposure in humans. The possibility of adenovirus and *Mycoplasma pneumoniae* that transmitted by the airborne route also warrants further investigation.

In this study, the detection rates of airborne adenovirus DNA products peaked in summer in the pediatric outpatient department and the pediatric emergency room, respectively. Furthermore, airborne *Mycoplasma pneumoniae* were frequently detected in the pediatric outpatient department and the detection rates were highest in autumn and winter seasons. This results suggest that the ventilation rate in pediatric public areas (including the emergency room and outpatient department) of hospitals should be increased during the peak seasons of adenovirus and *Mycoplasma pneumoniae* contamination to reduce the airborne bioaerosol levels. The limitation of this study was lack of environmental characteristics measurement along with airborne adenovirus and *Mycoplasma pneumoniae* sampling. Further study will enable the relationship between indoor environmental characteristics (including temperature, relative humidity and CO_2_ concentration) and the airborne adenovirus/*Mycoplasma pneumoniae* to be elucidated.

The air sampling time in the pediatric outpatient department was shorter than that in the pediatric emergency room. The main reason was that the pediatric outpatient department of the hospital was close at nighttime. The monthly detection rate of airborne adenovirus DNA products in the filter samples in the pediatric outpatient department was lower than that in the pediatric emergency room, but the measured viral concentrations in March, May, June, and July in the pediatric outpatient department considerably exceeded those measured in the pediatric emergency room. Additionally, *Mycoplasma pneumoniae* exhibited not only a higher detection rate but also higher concentrations in the pediatric outpatient department than in the pediatric emergency room. The possible cause may be the indoor unfair ventilation and more patients with *Mycoplasma pneumoniae* infection in the pediatric outpatient department. The results demonstrate that the mean CO_2_ concentration in the pediatric outpatient department exceeded that in the pediatric emergency room. Therefore, the ventilation system should be adjusted periodically and the air change rate should be increased during peak seasons with high viral concentrations in the pediatric public areas. Also, high-efficiency particulate air (HEPA) filters should perhaps be installed in the ventilation systems in the pediatric public areas of hospitals to maintain indoor air quality. In addition, the results indicated that monthly distributions of detection rates of adenovirus and *Mycoplasma pneumoniae* in the air samples were inconsistent with the median concentration distributions of airborne adenovirus and *Mycoplasma pneumoniae* in the pediatric public areas. The possible reason might be included: 1) the air samples in the pediatric public areas were only collected twice in a week; 2) the exact number of infected patients in the pediatric public areas was unknown.

A previous investigation found that the number of patients with influenza was closely correlated with the number of air samples that contained influenza A RNA (r = 0.77) [18]. Tseng et al. study indicated that the influenza A virus concentrations in the air were significantly correlated with the number of patients with low respiratory tract infections (r = 0.43, *p* = 0.02), but no correlation occurred between the viral concentrations and the number of patients with upper respiratory tract infections (r = 0.33, *p* = 0.06) [15]. During the study period, there was not outbreak occurrence of adenovirus and *Mycoplasma pneumoniae* infections in the hospital. There were a great number of patients and visitors in the hospital, and not all of patients with respiratory tract infection were tested for adenovirus or *Mycoplasma pneumoniae* infections, so it would be underestimated the number of patients with adenovirus or *Mycoplasma pneumoniae* infection in the pediatric public areas. The relationship between the number of infected patients and the number of air samples with detected certain pathogens warrants further investigation.

Indoor air quality is important to prevent infection and thereby protect patients and health care workers in hospitals. The quality of indoor air depends on heating ventilation and air conditioning (HVAC) systems, the number of persons, human activity and other factors (i.e., the outdoor air quality) [19], [20]. Until recently, no standard method had been established for detecting specific viral aerosol particles. Tolerable levels of airborne viruses in hospitals have not yet been established. Airborne particle concentration has been suggested to be an indicator of microbial contamination [21]. In this study, PM_10_ and PM_1_ levels were higher in the pediatric outpatient department than in the pediatric emergency room. Hence, further investigations must be performed to characterize the relationship between particulate matter levels, such as PM_10_ and PM_1_, and the concentrations of airborne viruses and bacteria.
